# Proposal for Using AI to Assess Clinical Data Integrity and Generate Metadata: Algorithm Development and Validation

**DOI:** 10.2196/60204

**Published:** 2025-06-30

**Authors:** Caroline Bönisch, Christian Schmidt, Dorothea Kesztyüs, Hans A Kestler, Tibor Kesztyüs

**Affiliations:** 1 Department of Electrical Engineering and Informatics, University of Applied Sciences Stralsund, Zur Schwedenschanze 15, Stralsund, 18435, Germany, 49 3831 45 6505; 2 Medical Data Integration Center Göttingen, University Medical Center Göttingen, Göttingen, Germany; 3 Institute of Medical Systems Biology, Universität Ulm, Ulm, Germany; 4 Medical Data Integration Center Ulm, Ulm, Germany

**Keywords:** metadata, quality, interoperability, reliability, utilization, AI, artificial intelligence, data integrity, algorithm, development, validation, clinical data, machine learning, literature review, accuracy, data quality, model

## Abstract

**Background:**

Evidence-based medicine combines scientific research, clinical expertise, and patient preferences to enhance the patient outcomes and improve health care quality. Clinical data are crucial in aligning medical decisions with evidence-based practices, whether derived from systematic research or real-world data sources. Quality assurance of clinical data, mainly through predictive quality algorithms and machine learning, is essential to mitigate risks such as misdiagnosis, inappropriate treatment, bias, and compromised patient safety. Furthermore, excellent quality of clinical data is a prerequisite for the replication of research results in order to gain insights from practice and real-world evidence.

**Objective:**

This study aims to demonstrate the varying quality of medical data in primary clinical source systems at a maximum care university hospital and provide researchers with insights into data reliability through predictive quality algorithms using machine learning techniques.

**Methods:**

A literature review was conducted to evaluate existing approaches to automated quality prediction. In addition, embedded in the process of integrating care data into a medical data integration center (MeDIC), metadata relevant to this clinical data was stored, considering factors such as data granularity and quality metrics. Completed patient cases with echocardiographic and laboratory findings as well as medication histories were selected from 2001 to 2023. Two authors manually reviewed the datasets and assigned a quality score for each entry, with 0 indicating unsatisfactory and 1 satisfactory quality. Since quality control was considered a binary problem, corresponding classifiers were used for the quality prediction. Logistic regression, k-nearest neighbors, a naive bayes classifier, a decision tree classifier, a random forest classifier, extreme gradient boosting (XGB), and support vector machines (SVM) were selected as machine learning algorithms. Based on preprocessing the dataset, training machine learning algorithms on echocardiographic, laboratory, and medication data, and assessing various prediction models, the most effective algorithms for quality classification were to be identified. The performance of the predictive quality algorithms was assessed based on accuracy, precision, recall, and scoring.

**Results:**

There were 450 patient cases with complete information extracted from the MeDIC data pool. The laboratory and medication datasets had to be limited to 4000 data entries each to enable manual review; the echocardiographic datasets comprised 750 examinations. XGB demonstrated the highest performance for the echocardiographic dataset with an area under the receiver operating characteristic curve (AUC-ROC) of 84.6%. For laboratory data, SVM achieved an AUC-ROC score of 89.8%, demonstrating superior discrimination performance. Finally, regarding the medication dataset, SVM showed the most balanced performance, achieving an AUC-ROC of 65.1%, the highest of all tested models.

**Conclusions:**

This proposal presents a template for predicting data quality and incorporating the resulting quality information into the metadata of a data integration center, a concept not previously implemented. The model was deployed for data inspection using a hybrid approach that combines the trained model with conventional inspection methods.

## Introduction

Evidence-based medicine combines the best available evidence from scientific research with clinical expertise and patient preferences. The goal of applying evidence-based medicine in health care is to enhance patient outcomes, improve the quality of care, and minimize ineffective or harmful treatments [1]. Valid clinical data and the associated metadata serve as the foundation for evidence-based medicine through high-quality evidence from systematic research or as real-world data (RWD) from clinical care [2]. It allows clinicians to align their decisions and treatments with the best available evidence from research and clinical guidelines, leading to better patient outcomes. Moreover, clinical data plays a crucial role in medical research and innovation. Researchers use large datasets to identify patterns, risk factors, and potential treatment approaches for various medical conditions, resulting in advancements and breakthroughs in medicine [3]. Without awareness of the data quality within these datasets, researchers and clinicians may face significant challenges, such as misdiagnosis and inappropriate treatment, compromised patient safety, and bias from confounding factors. In addition, the quality of data can impact the ability to replicate research results and affect the efficiency of data integration. To mitigate these risks, it is essential for clinicians and researchers to be mindful of the quality of the clinical data they are using [4].

Data integration centers, which are set up to consolidate clinical routine data in German university hospitals, can provide information about the quality of their data, given their extensive data resources [5]. The Medical Data Integration Center of the University Medical Center Göttingen (UMG-MeDIC) securely stores clinical data (eg, laboratory findings or echocardiography data) and makes it available upon request to enable as many researchers as possible to access the data while taking data protection and security into account [6].

Data originating from clinical care source systems that are not collected for the intended research use case are often incomplete, lack necessary information (eg, missing entries), or exhibit uncertain measurement quality [7]. Therefore, providing a precise and detailed description of data through metadata when merging heterogeneous sources enables a valid and meaningful integration aimed at ensuring traceable reuse. Metadata assists researchers and users in searching, categorizing, and interpreting data from a data warehouse. In addition, metadata ensures data consistency and facilitates data management [8,9].

Qualitative predictions using machine learning algorithms can be used to gather information about the completeness of data, which includes not only missing values but also semantic completeness and consistency. Machine learning and deep learning methodologies offer significant potential for ensuring quality. Nalbach et al [10] describe data-driven estimations of data quality as predictive quality. The extraction of data and its relation to quality metrics—such as completeness, consistency, correctness, and correspondence [11], enables data-driven quality assessment based on historical data. These estimations provide a foundation for decision-making regarding quality enhancement measures, such as informing data stewards about necessary missing values that should be added [10].

Based on the predictive quality approach further described by Tercan et al [12] and Schmitt et al [13], this research aimed to identify a predictive model-based quality algorithm for clinical data, including RWD, and provide automated quality inspection. The goal of this paper is to demonstrate the varying quality of medical data in primary clinical source systems and to inform researchers about data reliability using machine learning techniques. This approach seeks to identify predictive model-based quality algorithms for clinical data, including RWD, to facilitate automated quality inspection, ensuring data completeness and consistency. As a result of our proposal, we intend to enhance the reliability of clinical data used in evidence-based medicine and medical research.

## Methods

### Literature Review

A literature search regarding machine learning algorithms and data quality was conducted to provide an overview of the semantic completeness of data, consider previous work to build on existing knowledge, and classify this work’s results.

The literature review establishes the foundation for developing innovative approaches and solutions designed to assess the quality of clinical data within the UMG-MeDIC. The literature search was conducted in Embase via Ovid and PubMed and resulted in 118 search records. After removing duplicates, 104 results were included in the title and abstract screening. Following the elimination of publications unsuitable for the goals of this manuscript, 12 results remained. The full text of the identified articles was carefully reviewed, and valuable information was integrated into the introduction and methodology sections of this manuscript. The search strategy for PubMed and the corresponding PRISMA Flow Chart of the literature search are provided in Table 1 and Table 2 of the Multimedia Appendix 1 [1].

### Ethical Considerations

Ethical approval for the anonymized secondary health data analysis was obtained from the Ethics Review Committee of the University Medical Center Göttingen with the Ref. No. 21/9/18. All developments and experiments were performed in accordance with relevant guidelines and regulations. Furthermore, informed consent was obtained from all participants or their legal guardians and the original informed consent allows the secondary analysis without additional consent. Regarding the privacy and confidentiality protection of human participants all study data is deidentified.

### Prerequisites at the UMG-MeDIC

In the context of this project, it was decided to store all metadata linked to the primary clinical data from the source systems of the UMG in a relational database (MariaDB). This was considered necessary because the actual clinical data to which the metadata refers was already stored in the same relational database.

The meta and metadata were captured during routine care and originated from different hospital information systems, including clinical workstation systems, laboratory information systems, echocardiography systems, and microbiology systems.

Information from the specifications of the supplied data formats was extracted to develop the preparatory work for the quality assessment of the clinical data.

Moreover, the granularity of metadata extracted from the primary source systems was taken into account. Metadata in primary source systems is captured at varying levels of granularity (eg, metadata date as day-month-year hour-minute-second vs metadata date as month-year) and must therefore be included and processed accordingly.

Data quality was introduced as additional metadata in the relational database structure to quantify the quality of the UMG-MeDIC data.

### Data Preparation

**Figure 1.**
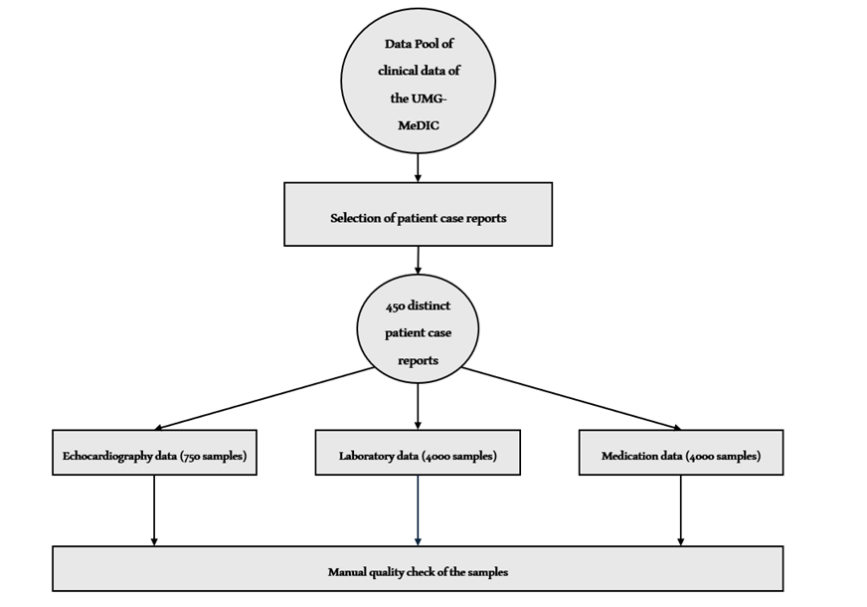
Outline of the data selection, starting from the data pool of clinical data from the UMG-MeDIC (Medical Data Integration Center of the University Medical Center Göttingen), where 450 patient case reports were selected manually. These 450 case reports were then divided into subclasses of echocardiographic data (sample size of 750), laboratory data (sample size of 4000), and medication data (sample size of 4000). Finally, the samples from each of the three subsets were manually checked for quality by two authors.

The clinical data, originating from the clinical source systems of the UMG, is heterogeneous. This data is extracted from various primary clinical sources, such as the lab information system and the clinical workstation system, and consolidated within the UMG-MeDIC. The data are pseudonymized during the consolidation and processing at the data integration center. Although the work is based on a technical viewpoint of the data sources, demographic information was not addressed separately. Patient cases were selected based on three criteria: (1) completed patient cases with (2) echocardiographic and laboratory findings, as well as medication histories (3) from 2001 to 2023, excluding data linked to outdated process configurations. Consequently, 450 distinct patient cases with clinical findings were extracted from the data pool of the UMG-MeDIC. Among these 450 case reports, each report includes echocardiographic, laboratory, and medication data as shown in Figure 1.

A total of 750 echocardiographic examinations were carried out for these 450 patient cases, which means around 1.6 echocardiographic examinations per patient case. In comparison, there was significantly more laboratory and medication data for these 450 patient cases, which did not allow a manual review of the quality of every individual data item. Hence, the laboratory and medication data subset was limited to 4000 data points each. On this basis, a dataset with 750 echocardiographic, 4000 laboratory, and 4000 medication data items was obtained.

Subsequently, 2 authors manually reviewed the echocardiographic, laboratory, and medication data, assigning each data entry a quality score from 0 to 1, where 0 indicates unsatisfactory quality and 1 indicates satisfactory quality.

The criteria for this assignment were based on corresponding preliminary work by the authors to identify quality measures [14] and further work related to quality metrics [11,15-17]. The first criterion is the semantic completeness of the data, a metric that assesses whether all mandatory data fields are filled with relevant information. Completeness ensures there are no missing values, enabling users to fully understand and use the data. The second measure, data consistency, evaluates whether the data complies with established standards, conventions, or formats. The third measure, data correctness, assesses the accuracy of the information. In addition, the linkage of this data to other datasets checks whether interlinked or interdependent data elements convey the same information across all instances. The quality metric data relevance ensures that the metadata meets the needs or expectations of its intended users. For example, if a dataset is designed for clinical research, including fields like measurement units might enhance its relevance. The metric semantic specificity considers the granularity and precision of semantic concepts in the data. High semantic specificity implies the use of detailed and well-defined terms to describe the information. Timeliness, the sixth metric, pertains to evaluating how current the data is. Timeliness is crucial, especially for dynamic or time-sensitive resources. The seventh metric, accessibility, measures whether the data is physically available and comprehensible to users (whether human or machine), while reproducibility of the data, the eighth and final metric, ensures that data evaluations are consistent and can be independently verified by different users or systems. After the separate reviews, the quality mappings were compared, and deviations were discussed. Ultimately, a uniform quality mapping for the echocardiographic, laboratory, and medication data was established.

This process ensures that the models exclusively learn from consistently occurring patterns and dependencies.

### Predictive Machine Learning Algorithms

Machine learning is a subfield of artificial intelligence (AI) that enables information technology systems to recognize patterns in existing data and develop solutions autonomously. This research involves classifying data quality variables for clinical data. The classification requires a training dataset with examples for each data source entity’s input and output variables.

All machine learning models and classifiers were developed in Python (Python Software Foundation) using scikit-learn libraries. Each machine learning algorithm was trained separately on echocardiographic, laboratory, and medication data to identify the best-performing algorithm for each dataset.

The machine learning algorithms selected include logistic regression (LR) [18], k-nearest neighbors (KNN) [19], a Naive Bayes (NB) classifier [20], a decision tree classifier [21], a random forest classifier [22], XGB [23], and support vector machines (SVM) [24].

For the echocardiographic data consisting of 750 samples, 575 samples have been classified. as unsatisfactory quality (0), while 175 samples are classified as good quality (1).

The laboratory data, comprising 4000 samples, revealed that 17 samples were classified as unsatisfactory (0) and 3983 samples were deemed to be of good quality (1).

The distribution of classes within the medication data resulted in 1280 samples of unsatisfactory quality (0) and 2720 samples of good data quality (1). Multimedia Appendix 2 provides an overview of the features for the three subsets and their target distribution. For each feature of the three subsets, the data are distributed across interval scales (Table 1).

The datasets exhibit an imbalanced target distribution, which necessitated consideration of the overclassification of the majority class. During data preprocessing, the Synthetic Minority Oversampling Technique (SMOTE) was used to tackle this imbalance. SMOTE generates synthetic data points by interpolating between minority class samples. The sampling strategy in SMOTE was set to default (auto), with a random state of 0 and k-neighbors configured to 5.

**Table 1.** Depiction of the features within the three subsets (echocardiography, laboratory and medication) with the data type of the feature and the number of features listed.

#Numbers	Column	Data type
Echocardiography		
0	untersuchungsalter	Int64
1	groesse	Float64
2	gewicht	Float64
3	aoroot	Float64
4	lads	Float64
5	ivsd	Float64
6	lvdd	Float64
7	lvpwd	Float64
8	tapse	Float64
9	lfev	Float64
10	lvef_vis	Float64
11	lvds	Float64
12	lvedv	Float64
13	lvesv	Float64
14	ladslong	Float64
15	laflaeche	Float64
16	lavolindex	Float64
17	mvevmax	Float64
18	quality	Int64
Laboratory		
0	Vorgang_Ref	Int64
1	Probe_Ref	Int64
2	Ergebnis_ID	Int64
3	Analyse_Typ_ID	Int64
4	Ergebnis_Wert	Float64
5	Ergebnis_Darstellung	Float64
6	Ergebnis_Manuell	Float64
7	Inaktiv	Int64
Medication		
0	application_type	Int64
1	application_deleted	Int64
2	application_bolus_value	Float64
3	application_dose_value	Float64
4	application_perfusor_volume	Float64
5	application_route	Int64
6	application_site	Float64
7	application_status	Int64
8	external_id.1	Int64
9	mid.1	Int64
10	active	Int64
11	additional_fee_obligation	Int64
12	availability	Int64
13	charge_documentation_required	Int64
14	out_of_trade	Int64
15	recent	Int64
16	reference_value	Float64
17	source_id	Int64
18	successor_id	Float64
19	version	Int64
20	external_id.2	Int64
21	amount	Float64
22	ingredient_type	Float64
23	main_ingredient_id	Float64
24	sequence_number	Int64
25	meona_substance_id	Float64
26	external_id.3	Int64

For input feature extraction, a multilayer perceptron autoencoder model was used, where each layer of the encoder uses batch normalization and leaky ReLU activation. The autoencoder was chosen because it automatically learns feature representations, which reduces the need for explicit feature selection methods that involve extensive iterative training. In addition, the ability to handle nonlinear relationships within the data necessitated the use of an autoencoder. Finally, the datasets were divided into training and test sets and validated using 5x10-fold cross-validation.

Since it is not feasible to universally preselect suitable algorithms in advance, it was necessary to test and evaluate various learning algorithms tailored to specific scenarios [25].

The LR algorithm was selected as the baseline for classification tasks because it predicts the probability of a target variable and is widely used in both binary and multiclass classifications. The parameters included the solver (newton-cg, lbfgs, and liblinear), a default penalty, and the inverse of regularization strength (C) set as loguniform (1e-5, 100).

The KNN classifies a data point based on the majority class among its k nearest neighbors. Due to its simplicity and effectiveness for low-dimensional data, it is a strong candidate for a benchmark. The parameters are as follows: “n_neighbors” ranges from 1 to 30 in increments of 5; weights can be uniform; the algorithm options include "ball_tree, kd_tree, and brute;” "leaf_size” can be 1, 10, or 30; and p is 1 for manhattan distance and 2 for euclidean distance.

NB applies Bayes’ theorem under strong independence assumptions. It is computationally efficient and performs effectively with high-dimensional data. The smoothing parameter prevents zero probabilities for unseen data, so it was set to alpha: 0.01 to 10.0.

Decision trees split data hierarchically based on feature thresholds to predict target labels. Hyperparameters like depth and minimum samples help reduce overfitting with this algorithm. In addition, decision trees are interpretable and can handle nonlinear relationships. The parameters set were “max_depth”: 3 or none, “max_features”: randomized range, “min_samples_leaf”: randomized range, and criterion: gini or entropy.

While random forests are resilient to overfitting and excel across various datasets, parameter tuning aims to balance tree diversity with computational cost. The parameters include bootstrap: true, “max_depth”: the maximum depth of trees (10, 20, 50, 100, or None), “max_features”: the number of features considered (auto or sqrt), “min_samples_leaf”: options of 1, 2, or 4, “min_samples_split”: the minimum number of samples needed to split a node (2, 5, or 10), and “n_estimators”: the total number of trees in the forest (50 or 100).

XGBoost is an optimized gradient-boosting algorithm that iteratively improves model predictions. It is renowned for its performance with structured or tabular datasets, and through hyperparameter tuning, it seeks to minimize overfitting while boosting prediction accuracy. The parameters include “learning_rate”: 0.05 to 0.30, “max_depth”: the maximum depth of the trees (3 to 15), “min_child_weight”: the minimum sum of instance weights in a child node (1 to 7), gamma: the minimum loss reduction required to make a split (0.0 to 0.4), and “colsample_bytree”: the fraction of features sampled for each tree (0.3 to 0.7).

As a large margin classifier, the SVM identifies a hyperplane that best separates classes in high-dimensional space. While it is effective for both linear and nonlinear data, a linear kernel was used here for computational efficiency, given the dataset size (kernel: linear kernel for simplicity and interpretability; probability: enabled for AUC-ROC computation).

## Results

### Literature Review Overview

The literature search yielded valuable insights into the relationship between machine learning algorithms and data quality, specifically focusing on semantic completeness within clinical datasets. After applying the screening process, the 12 retained articles provided key perspectives on data quality assessment, machine learning methodologies, and their application within clinical environments. A thorough analysis of these studies revealed several recurring themes. First, a significant portion of the literature addressed the challenges of ensuring semantic completeness in structured and unstructured clinical data. Several studies emphasized the necessity of standard terminologies and ontologies to enhance data consistency and interoperability. In addition, multiple sources explored different machine learning techniques, such as supervised and unsupervised learning approaches, to detect and mitigate issues related to incomplete or inconsistent data. Key insights from the literature were incorporated into the introduction and methodology sections, providing a solid theoretical foundation for this study. The findings underscored the importance of leveraging machine learning models to improve clinical data integrity and supported the need for robust evaluation metrics to quantify data quality improvements. The PRISMA (Preferred Reporting Items for Systematic reviews and Meta-Analyses) Flow Chart and detailed search strategy, presented in Table S2 of Multimedia Appendix 1, further outline the systematic approach used in identifying relevant studies.

Since here we consider quality prediction as a binary classification problem, we evaluated binary-class classifiers on the provided datasets. We then compared the results of each model in a table that included accuracy, precision, recall, and scoring.

### Predictive Machine Learning Algorithms

Multiple machine learning algorithms were evaluated for predicting the quality of echocardiography data, with LR serving as the baseline model. LR achieved an accuracy of 73.0% (0.73/1), precision of 57.1% (0.571/1), recall of 73.0% (0.73/1), F_1_-score of 64.1% (0.641/1), and an AUC-ROC score of 52.4% (0.524/1). While this model provides a foundational benchmark, other algorithms demonstrated superior performance across all metrics, suggesting their greater suitability for this dataset, as shown in Table 2.

KNN demonstrated improvements over LR, achieving an accuracy of 76.6% (0.766/1) and an AUC-ROC score of 70.3% (0.703/1). The improved precision (0.744/1, 74.4%) and F_1_-score (0.684/1, 68.4%) emphasize its more effective balance of false positives and false negatives compared to the baseline.

NB attained a recall rate of 75.9% (0.759/1), comparable to KNN, but exhibited relatively lower precision at 57.6% (0.576/1) and an AUC-ROC score of 51.0%. This suggests a trade-off in its predictive performance.

The decision tree classifier slightly outperformed LR, reaching an accuracy of 73.7% (0.737/1) and an AUC-ROC score of 71.3% (0.713/1). The F_1_-score of 73.7% (0.737/1) emphasizes its ability to maintain a balanced level of precision and recall.

The random forest classifier delivered a notable improvement, achieving an accuracy of 82.5% (0.825/1) and an AUC-ROC score of 85.3% (0.853/1). These metrics, alongside an F_1_-score of 81.0% (0.81/1), highlight its robust performance and effectiveness in capturing complex patterns in the data.

XGB achieved the highest overall performance, with an accuracy of 84.7% and an AUC-ROC score of 84.6%. Its F_1_-score of 84.0% and precision of 83.9% demonstrate its ability to consistently deliver high-accuracy predictions while minimizing false positives.

The SVM achieved an accuracy of 73.0%, comparable to that of LR, but it demonstrated an improvement in the AUC-ROC score of 65.7%. Its F_1_-score of 67.1% reflects a slight enhancement in predictive balance.

The laboratory dataset consisted of 4000 data items. The performance of the machine learning algorithms was evaluated on the laboratory dataset, again using LR as the baseline model (refer to Table 3). LR achieved an accuracy of 99.8%, a precision of 99.5%, a recall of 99.8%, an F_1_-score of 99.6%, and an AUC-ROC score of 33.1%. Although LR demonstrated strong results in accuracy, precision, and recall, its relatively low AUC-ROC score indicates limitations in its ability to effectively discriminate between classes.

**Table 2.** Scoring of the prediction classifiers (in percent) evaluated for the echocardiographic data set with Logistic Regression, K-Nearest Neighbors, Bayes Classifier, Decision Tree, Random Forest, Extreme Gradient Boosting, and SVM. While XGB’s accuracy is 84.7, the AUC-ROC score is 84.6. In comparison, the accuracy of the RF is 82.5, but the AUC-ROC score is 85.3.

	Accuracy	Precision	Recall	F_1_-score	AUC-ROC^a^ score
Logistic regression	73.0	57.1	73.0	64.1	52.4
K nearest neighbors	76.6	74.4	76.6	68.4	70.3
Naive Bayes classifier	75.9	57.6	75.9	65.5	51.0
Decision tree classifier	73.7	73.7	73.7	73.7	71.3
Random forest classifier	82.5	81.3	82.5	81.0	85.3
Extreme gradient boosting	84.7	83.9	84.7	84.0	84.6
Support vector machine	73.0	65.2	73.0	67.1	65.7

a AUC-ROC: area under the receiver operating characteristic curve.

**Table 3.** Scoring of the prediction classifiers (in percent) evaluated for the laboratory data set with Logistic Regression, K-Nearest Neighbors, Bayes Classifier, Decision Tree, Random Forest, Extreme Gradient Boosting, and SVM. While the accuracy of all classifiers is nearly the same, SVM outperforms with the highest AUC-ROC score of 89.8.

	Accuracy	Precision	Recall	F_1_-score	AUC-ROC^a^ score
Logistic regression	99.8	99.5	99.8	99.6	33.1
K nearest neighbors	99.9	99.9	99.9	99.9	74.6
Naive Bayes classifier	99.8	99.5	99.8	99.6	50.0
Decision tree classifier	99.8	99.5	99.8	99.6	79.2
Random forest classifier	99.9	99.9	99.9	99.9	74.2
Extreme gradient boosting	99.5	99.7	99.5	99.6	59.3
Support vector machine	99.8	99.5	99.8	99.6	89.8

a AUC-ROC: area under the receiver operating characteristic curve.

KNN slightly outperformed LR in accuracy (0.999/1, 99.9%) and recall ( 99.9%). Its AUC-ROC score of 74.6% (0.746/1) indicates a notable enhancement in classification discrimination compared to LR.

NB achieved results similar to LR, with an accuracy of 99.8% (0.998/1) and an AUC-ROC score of 50.0% (0.5/1). Although its precision, recall, and F_1_-score matched those of LR, the modest AUC-ROC score indicates potential limitations in distinguishing between classes in certain situations.

DT matched the accuracy, precision, and recall of LR but demonstrated a marked improvement in its AUC-ROC score (0.792/1, 79.2%). This highlights its enhanced ability to effectively separate classes despite similar performance on other metrics.

RF also achieved 99.9% (0.999/1) accuracy, precision, and recall, equaling KNN in these metrics. However, its AUC-ROC score of 74.2% (0.742/1) suggests that its performance in classification discrimination is slightly less robust than that of DT but still superior to LR.

XGB achieved a high accuracy of 99.5% (0.995/1) and an F_1_-score of 99.6% (0.996/1). However, its AUC-ROC score of 59.3% (0.593/1) reveals its lower discrimination capacity compared to DT, RF, and KNN in this dataset.

SVM achieved strong results across all metrics, with accuracy, precision, recall, and F_1_-score aligning with those of the top-performing models (99.8%‐99.9%). Notably, its AUC-ROC score of 89.8% (0.898/1) is the highest among all models, demonstrating superior discrimination performance within this dataset.

For the medication dataset, a sample of 4000 data items was cleaned and analyzed regarding the target quality. As shown in Table 4, the baseline model LR achieved an accuracy of 59.2% (0.592/1), precision of 35.0% (0.35/1), recall of 59.2% (0.592/1), an F_1_-score of 44.0% (0.44/1), and an AUC-ROC score of 45.8% (0.458/1). Although it performed better than several models in terms of recall and accuracy, its overall low precision and AUC-ROC score indicate limited effectiveness in distinguishing between classes.

KNN performed lower than LR, with an accuracy of 48.3%, precision of 43.8%, and a slightly better F_1_-score of 44.9%. However, its AUC-ROC score of 43.2% was lower, indicating weaker discriminatory ability compared to LR.

NB showed results similar to LR, achieving an accuracy of 58.3% and an F_1_-score of 43.6%. However, its AUC-ROC score of 42.6% indicates slightly lower classification discrimination compared to LR.

DT achieved an accuracy of 51.7%, a precision of 39.5%, and a recall of 51.7%, resulting in an F_1_-score of 42.5%. Its AUC-ROC score of 43.1% was comparable to that of KNN and NB, but slightly lower than LR, indicating similar performance levels.

RF and XGB performed poorly on this dataset. RF achieved an accuracy of 30.0%, an F_1_-score of 29.5%, and an AUC-ROC score of 22.2%. XGB exhibited even lower accuracy at 21.7%, with precision at 23.3%, an F_1_-score of 22.3%, and an AUC-ROC score of 19.0%, underscoring the significant limitations of these models for this dataset.

The SVM exhibited the most balanced performance among the models, achieving an AUC-ROC score of 65.1%, the highest across all models. Although it has a relatively low accuracy of 30.0% and an F_1_-score of 30.0%, its AUC-ROC score suggests strong potential for distinguishing between classes.

Based on the results of testing different machine learning algorithms on the three datasets, the XGB model was deployed for echocardiographic data, the SVM for laboratory data, and the SVM for medication data during the UMG-MeDIC data inspection process.

The data inspection process used a hybrid approach, meaning that data quality inspection was not solely reliant on the trained model; conventional inspections were also conducted. To achieve this, it was decided that data items flagged as unsound during quality prediction would undergo traditional inspection to reduce false-positive rates and fine-tune the models. The classifiers were then retrained with the new test data.

**Table 4.** Scoring of the prediction classifiers evaluated for the medication data set with Logistic Regression, K-Nearest Neighbors, Bayes Classifier, Decision Tree, Random Forest, Extreme Gradient Boosting and SVM. While LR and NB performed with the best accuracy (59.2, 58.3), SVMs AUC-ROC score of 65.1 was the highest.

	Accuracy	Precision	Recall	F1-score	AUC-ROC score
Logistic regression	59.2	35.0	59.2	44.0	45.8
K nearest neighbors	48.3	43.8	48.3	44.9	43.2
Naive Bayes classifier	58.3	34.8	58.3	43.6	42.6
Decision tree classifier	51.7	39.5	51.7	42.5	43.1
Random forest classifier	30.0	29.0	30.0	29.5	22.2
Extreme gradient boosting	21.7	23.3	21.7	22.3	19.0
Support vector machine	30.0	32.1	30.0	30.0	65.1

### Prerequisite at the UMG-MeDIC

**Figure 2.**
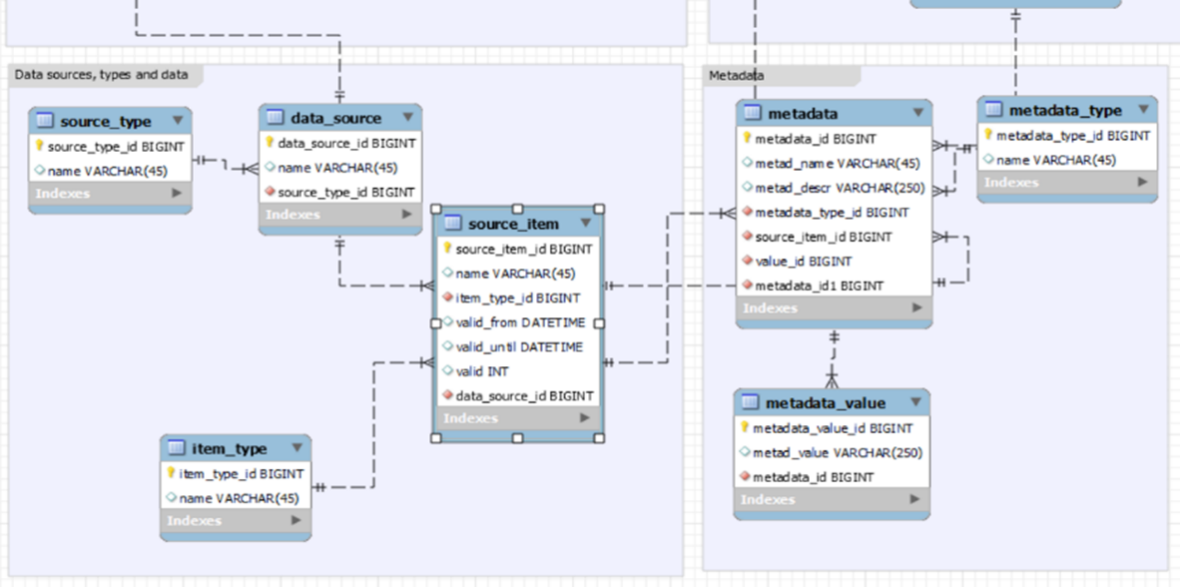
Relational structure and the metadata tables within the UMG-MeDIC (Medical Data Integration Center of the University Medical Center Göttingen).

In general, predicting the data quality for echocardiography, laboratory, and medication data is now possible based on trained ML models, allowing the results to be made available to researchers. The relational structure established for metadata stores information in the metadata table, which includes an ID, the name of the metadata, a description of the metadata, and details about “metadata_type_id,” “source_item_id,” and “value_id,” all of which are inherited from other tables through foreign keys in the relational structure. The respective metadata value is stored in the metadata_value column of the metadata_value table, as illustrated in Figure 2.

The predictive quality information is integrated via SQL queries into the relational database within UMG-MeDIC. Specifically, the metadata for the predictive quality data is stored in the metadata table, while the corresponding results of the predictive quality analysis, in terms of actual values, are stored in the “metadata_value” table. This setup enables easy retrieval and analysis of the prediction results, as the metadata table acts as the central reference point for all integrated data, with each entry linking back to its respective predicted values in the metadata_value table. Figures 8 and 9 (second reference in Multimedia Appendix 1 [2]) illustrate an example of the metadata structure within the relational database that includes the added predictive quality information.

## Discussion

### Conclusion

The benefits of RWD for medical care and research are numerous, ranging from clinical decision-making and patient-centered medicine to its application as synthetic controls in randomized trials. Moreover, real-world evidence enhances randomized controlled trials to bridge the efficacy-effectiveness gap. The central requirement for leveraging the data to achieve these advantages is data quality, particularly its reliability [26]. The quality of the data can be articulated or supported with corresponding metadata, thus further investigation into predicting data quality with AI is necessary. Given the significant importance of reliable health care data for researchers in reusing it, the UMG-MeDIC has incorporated metadata on data quality as easily accessible information.

### Principal Findings

All previously collected metadata concerning the primary source systems of the UMG within CouchDB were extracted and transferred to the newly established generic relational metadata structure within the data warehouse (DWH), as described in Section Prerequisite at UMG-MeDIC. This relational structure aligns with the DWH’s underlying design and serves as another principal finding of this work. After the algorithm transferred all existing metadata into the newly created relational metadata tables, the next step was to adjust the extract-transform-load (ETL) processes. In addition, metadata types, such as predicted data quality and data item language, which had not been previously collected and stored, are now being integrated into the existing metadata, providing more information about data reliability.

Based on the above results, predicting data quality for the 3 clinical datasets served as a case study. It evaluated the approach of predicting data quality within the ETL process, storing the resulting data quality as metadata alongside other metadata, and making the information available for researchers.

The study evaluated several machine learning models for predicting data quality across 3 different datasets: echocardiographic, laboratory, and medication data. The results of the echocardiographic data show that ensemble methods like RF and XGB outperform simpler models, including the baseline model LR and NB. The strong AUC-ROC scores of RF and XGB highlight their ability to effectively discriminate between classes, making them the most suitable models for this dataset.

While most models demonstrated high accuracy, precision, recall, and F_1_-scores for predicting the laboratory data, significant variations were observed in AUC-ROC scores. SVM emerged as the most effective model for this dataset, combining high overall performance with the best class discrimination capabilities. KNN, DT, and RF also performed well, showing notable improvements over LR regarding AUC-ROC scores.

The medication dataset showed lower performance across all models. LR outperformed other models in terms of accuracy and recall, making it the most effective choice for this dataset. However, SVM’s superior AUC-ROC score suggests it may be better suited for tasks requiring high class separation. Models like RF and XGB underperformed significantly, possibly due to the characteristics of this dataset in particular. This indicates a poor fit without further data engineering.

### Comparison to Previous Work

To the best of our knowledge, data quality prediction based on specific quality factors for echocardiographic, laboratory, and medication data, as well as the usage and provision of the resulting data quality within metadata, have not yet been implemented.

Therefore, the approach taken in this work is aligned with similar studies in other fields. Schmitt et al describe a comparable procedure concerning the inspection process in the electronic industry for producing programmable logic controllers. The predictive model-based quality inspection framework provided there was adapted for application in the Medical Data Integration Center.

Regarding metadata, no comparable examples were found in the field of medical data. However, a similar method for assessing the quality of medical data was employed by Haghighat et al for digitized histological glass slides, where they also used AI to evaluate the utility and diagnostic level of these images [27]. According to Georgiev and Valkanov, the future of DWH data quality, however, lies in further developing ETL processes towards dynamic and adaptable solutions with automated identification and elimination of quality issues based on artificial intelligence [28].

The existing metadata needed to be transferred and stored in the correct table at the right location. This process only had to be done once for slightly over 3,000 existing metadata documents. From now on, all additional metadata will be loaded directly into the relational metadata structure through adapted ETL routes. In contrast, Oukhouya et al propose a generic metadata management model designed specifically for data from heterogeneous sources to be stored in a DWH [29]. Their model is based on a data lake, a concept introduced in business intelligence architecture to manage large volumes of unstructured and semistructured data. They identified several essential functionalities of metadata that are critical for supporting a metadata management system. Requirements may vary significantly between DWHs in the medical field and those in other sectors. Nonetheless, this comprehensive model could serve as a starting point for developing suitable metadata management for medical DWHs to avoid reinventing the wheel. Therefore, in further developing metadata management for the DWH of UMG MeDIC, the extent to which the proposed model can provide applicable information for a relational database system should be evaluated.

### Strengths and Limitations

First, the low performance of machine learning models, particularly in echocardiographic and medication samples, could be due to several factors. Models may struggle if the features in the dataset lack sufficient discriminatory power or if the presence of irrelevant or inconsistent data impairs their ability to learn effectively.

To address these issues, an error analysis was conducted to identify which classes are most often misclassified. Therefore, confusion matrices were used to identify false positives and false negatives for each model (see third reference in Multimedia Appendix 1 [3]).

Second, the high occurrence of false negatives in the LR, KNN, and NB indicates that the data within the medication dataset are composed of non-linear relationships. Furthermore, the high dimensionality (many features) of the medication dataset results in problematic KNN calculations. The confusion matrix for the DT shows that the model struggles to correctly identify class 0. It almost always predicts class 1, even when class 0 would be the correct prediction. To improve this behavior, we performed a threshold value optimization. The XGB model demonstrates significant weaknesses in class differentiation, particularly for class 0. A potential reason for this issue may be that the features lack sufficient discriminatory power. The same observation applies to RF and SVM.

The performance of the models within the echocardiography dataset indicates that the dataset includes non-linear relationships and dependencies among the features. The slightly higher performance of models like RF and XGB also suggests that non-linear relationships play a role.

Third, 750 data points may not be sufficient for models like RF, XGB, and SVM, which typically learn from a large number of parameters and data points. These models often require more data to generalize effectively.

Although the medication dataset model currently shows lower predictive performance (accuracy: 30%, AUC-ROC: 65.1%), it has been integrated into the data inspection process as part of a hybrid approach, where automated predictions enhance traditional inspection methods. Rather than replacing manual review, the model assists in prioritizing data items for further inspection, enabling data stewards to concentrate on potentially lower-quality records.

Continual model retraining poses challenges, including catastrophic forgetting, data drift, and a need for robust evaluation strategies [30,31]. To tackle these issues, the model undergoes scheduled retraining with updated data while performance is monitored using metrics such as accuracy, recall, and AUC-ROC. The impact of human factors in creating, consolidating, and evaluating the database, especially regarding potential errors, must be taken into account. Moreover, it can be assumed that an increased number of connected systems in a MeDIC will reduce human errors but also create new types of errors [32].

Thus, newly flagged data points undergo manual verification before being included in future training iterations, ensuring that retraining enhances rather than diminishes performance. In productive operation, new, realistic data is often continuously available, better reflecting how the target variables behave in real life. Additionally, models such as RF, XGB, and SVM significantly benefit from a larger database, allowing them to learn more reliable patterns.

The machine learning algorithms ran on a laptop with Microsoft Windows 11 Pro, which features an Intel Core processor with 4 cores. The prediction runtime was about 10 minutes for all the algorithms. The models are currently deployed at UMG-MeDIC, and there is an inspection strategy in place that follows a hybrid approach.

However, it is important to note that the technical implementation of the relational data structure depends on the inherent and specific requirements of the UMG-MeDIC. This means that while the data storage is specialized, the quality prediction of the clinical data remains unaffected by it.

Nevertheless, scalability for datasets containing vastly different types of clinical data, such as imaging data or genomic sequences, may require additional preprocessing or adaptation steps. For instance, the features extracted from such data may vary significantly from those used in the current work, necessitating customized data preparation pipelines and potentially adjusted analytical models. Furthermore, the quality scale employed was established as binary, using 0 and 1; a more refined granularity of the quality scale is pending further development.

### Future Directions

Future work aims to enhance model performance by applying feature engineering, using data augmentation techniques, and investigating possible adjustments in model selection. Despite current limitations, even imperfect predictive models are valuable as they direct manual review efforts, reduce workload, and improve overall data quality assessment.

Furthermore, it must be discussed to what extent the metadata published in the different data formats contain the risk of reidentification. Metadata do not intrinsically contain any personal data within the UMG-MeDIC. However, it must be examined how far the linkage of the metadata with further information or the uniqueness of the metadata increase the risk of re-identification.

The ability to provide researchers with quality information on data they want to inspect and research is a key success factor for reliable medical and biomedical research, avoiding the “garbage in—garbage out” paradigm [33]. Due to the abundance of data accessible in a medical data integration center, employing predictive model-based quality inspection shows great promise in ensuring reliable data for medical research.

However, real-world deployment brings additional challenges such as data drift, generalizability across clinical settings, and resilience against distribution shifts. Additionally, it’s important to note that as more systems connect within a MeDIC, human errors (eg, transmission errors) decline, yet new types of errors may arise, which are part of the continuous learning process of these systems.

Moving forward, it is necessary to conduct a postdeployment evaluation, which includes the monitoring of prediction performance on live data at the UMG-MeDIC data integration center, comparing model outputs with manual quality assessments from data stewards, testing on external datasets to ensure generalizability beyond internal sources, and longitudinal tracking of model performance to detect degradation over time.

This expanded evaluation will allow us to refine model performance, improve generalization, and provide a more comprehensive assessment of real-world impact. By integrating these additional validation steps, we aim to ensure the long-term reliability and effectiveness of our approach in clinical data quality assessment.

## Supplementary material

10.2196/60204 Multimedia Appendix 1 Depiction of Supplementary Material including additional data, figures, and detailed methods supporting the findings presented in the main manuscript.

10.2196/60204 Multimedia Appendix 2 Comparison of the target distribution within the three subsets of echocardiographic data(750 samples), laboratory data (4000 samples) and medication data (4000 samples). The dark grey category comprises data samples with satisfactory quality (quality = 1) and the light grey category depicts data with unsatisfactory quality (quality = 0).

## Abbreviations

AI: artificial intelligence
AUC-ROC: area under the receiver operating characteristic curve
DWH: data warehouse
ETL: Extract-Transform-Load
KNN: k-nearest neighbor
LR: logistic regression
MeDIC: Medical Data Integration Center
NB: Naive Bayes
PRISMA: Preferred Reporting Items for Systematic reviews and Meta-Analyses
RWD: real world data
SMOTE: Synthetic Minority Oversampling Technique
SVM: support vector machine
UMG: University Medical Center Göttingen
XGB: extreme gradient boosting
