# Supplementary material for: Proposal for Using AI to Assess Clinical Data Integrity and Generate Metadata: Algorithm Development and Validation
Source: JMIR Med Inform. 2025 Jun 30;13:e60204. doi: 10.2196/60204 (PMC12234397; doi:10.2196/60204)
Supplement: Multimedia Appendix 1 [file medinform-v13-e60204-s001.pdf]

Supplementary Materials

[1]

Number	Search Step	Results
1	"Data Management" [MeSH Terms] OR "Data Curation"[MeSH Terms] OR "Data Accuracy"[MeSH Terms] OR "data quality"[Title/Abstract] OR "data reliability" [Title/Abstract: ~5]	29,298
2	"metadata"[MeSH Terms] OR "metadata quality"[Title/Abstract: ~5]	854
3	"Data Warehousing"[MeSH Terms] OR "data warehouse"[Title/Abstract] OR "research data"[Title/Abstract] OR "healthcare data"[Title/Abstract]	14,317
4	"Algorithms"[Mesh] AND ("Machine Learning"[Mesh] OR "Artificial Intelligence"[Mesh]) OR (("machine learning"[Title/Abstract] OR "artificial intelligence"[Title/Abstract]) AND "algorithm"[Title/Abstract])	223,743
5	(#1 OR #2) AND (#3 AND #4)	72

Table 1 Exemplary representation of the literature search steps including search terms, the connection between the the search terms and the corresponding results conducted within PubMed.

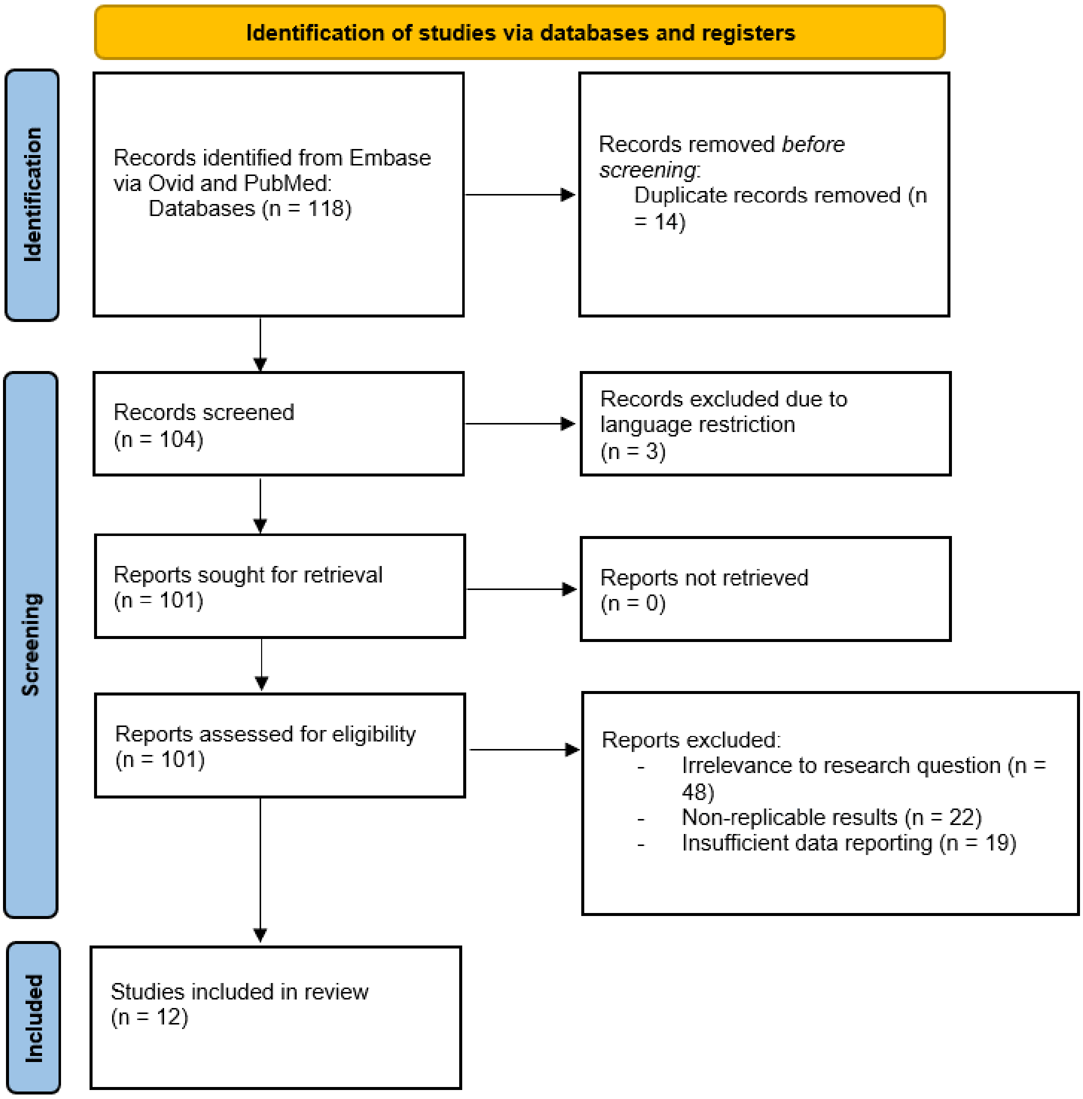

Table 2 Based on the literature search the process of identification, screening and inclusion is shown in the PRISMA Flow chart [35].

[2]

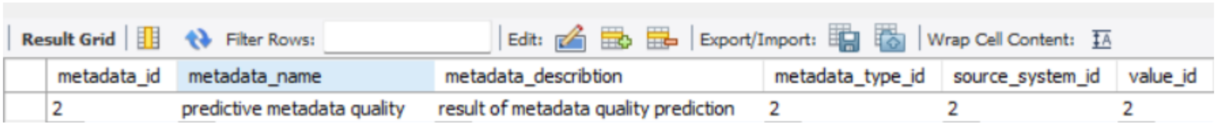

metadata_id	metadata_name	metadata_describtion	metadata_type_id	source_system_id	value_id
2	predictive metadata quality	result of metadata quality prediction	2	2	2

Figure 8. Examplary depiction of the predictive metadata quality within the metadata table in the MariaDB of the UMG-MeDIC. In this example the metadata of the predictive quality refers to the medication information system of the UMG as referenced in the source_id = 2.

	metadata_value_id	metadata_value	metadata.metadata_id
	2	1	2
	3	0	2
	4	0	2
	5	0	2
	6	1	2
	7	0	2
	8	0	2
	9	0	2
	10	0	2
	11	1	2
	12	1	2
	13	1	2

Figure 9. Exemplary depiction of the actual results of the metadata quality prediction, as stored in the metadata_value table of the UMG-MeDIC.

[3]

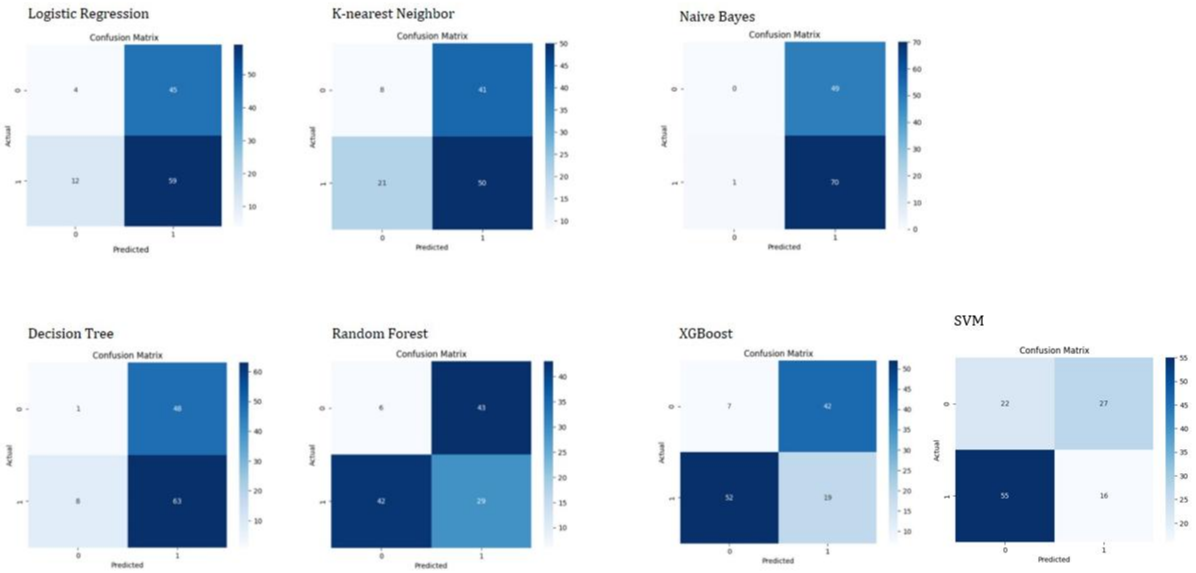

Figure 10. Representation of the confusion matrices of the different machine learning models LR, KNN, NB, DT, RF and XGB for the medication dataset.

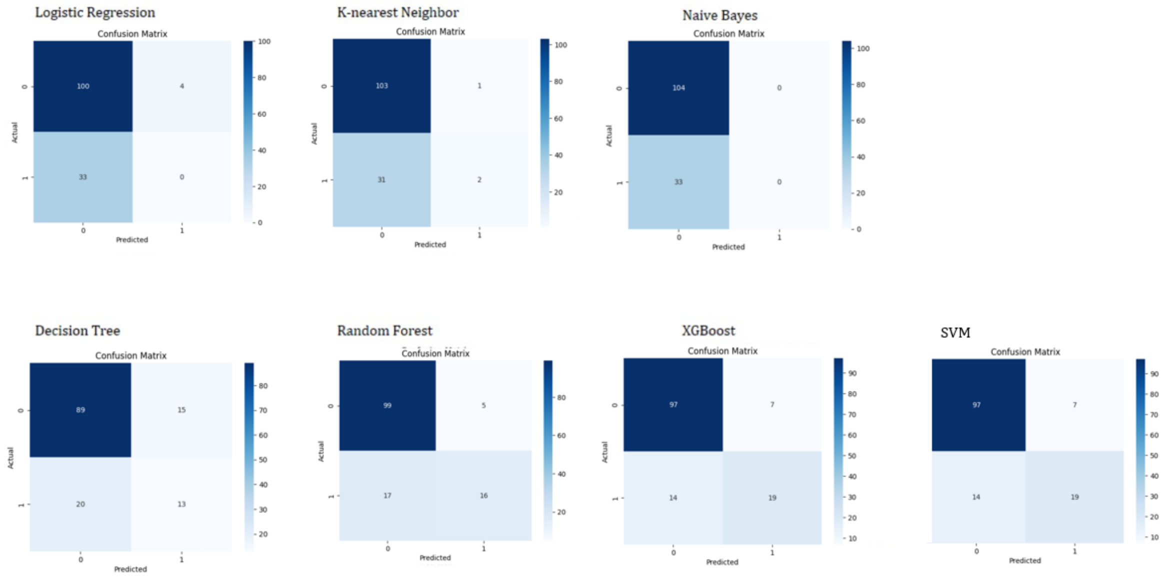

Figure 11. Representation of the confusion matrices of the different machine learning models LR, KNN, NB, DT, RF, XGB and SVM for the echocardiography dataset.

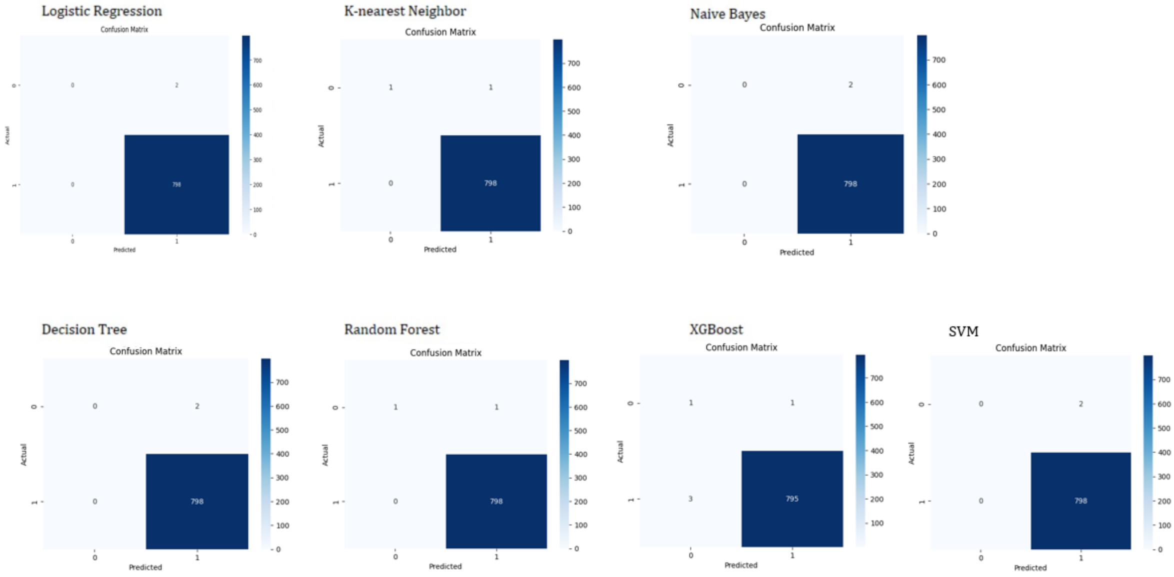

Figure 12. Representation of the confusion matrices of the different machine learning models LR, KNN, NB, DT, RF, XGB and SVM for the echocardiography dataset.

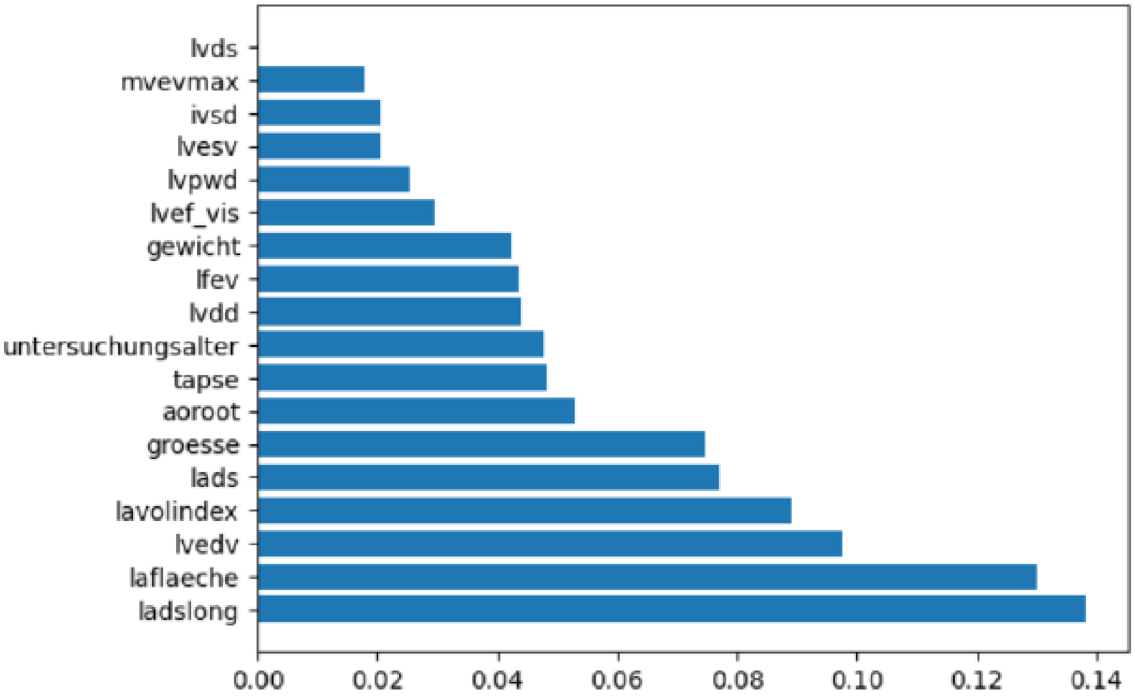

Figure 13. Random Forest relevance of features of the echocardiographic dataset

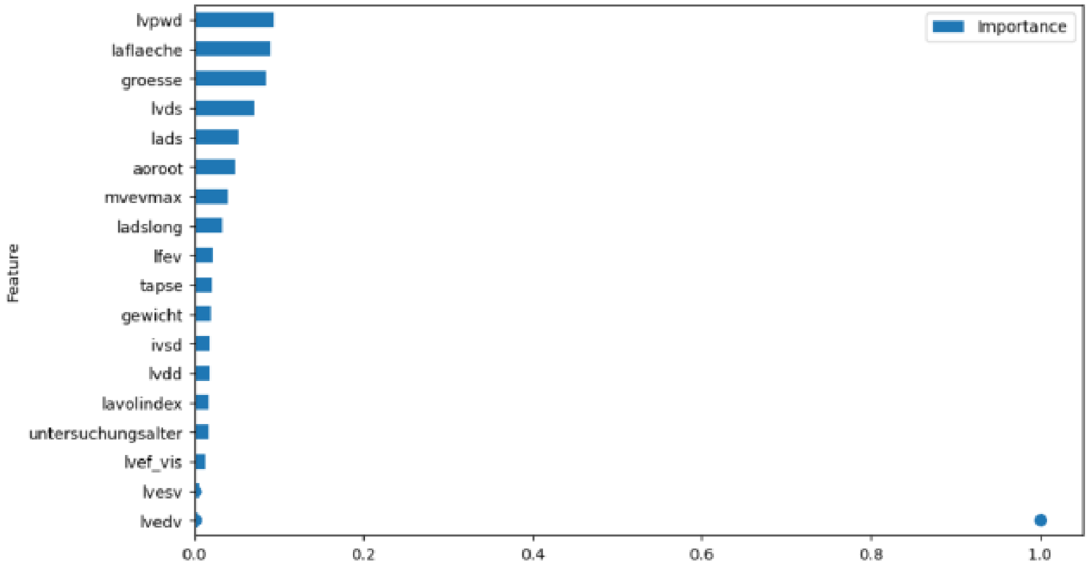

Figure 14. LR relevance of features of the echocardiographic dataset

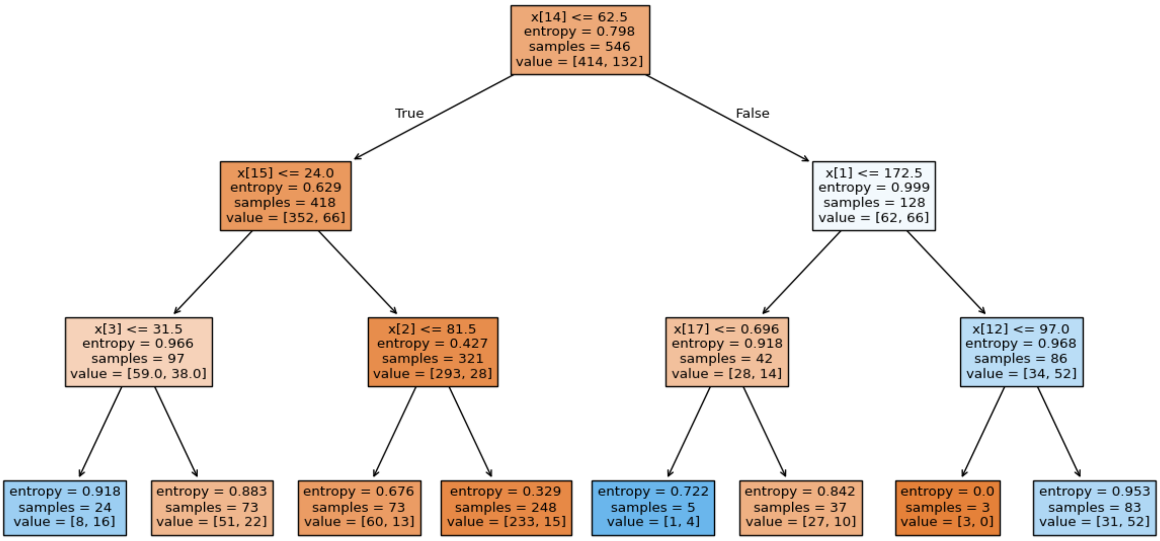

Figure 15. Results of the DT of the echocardiographic dataset

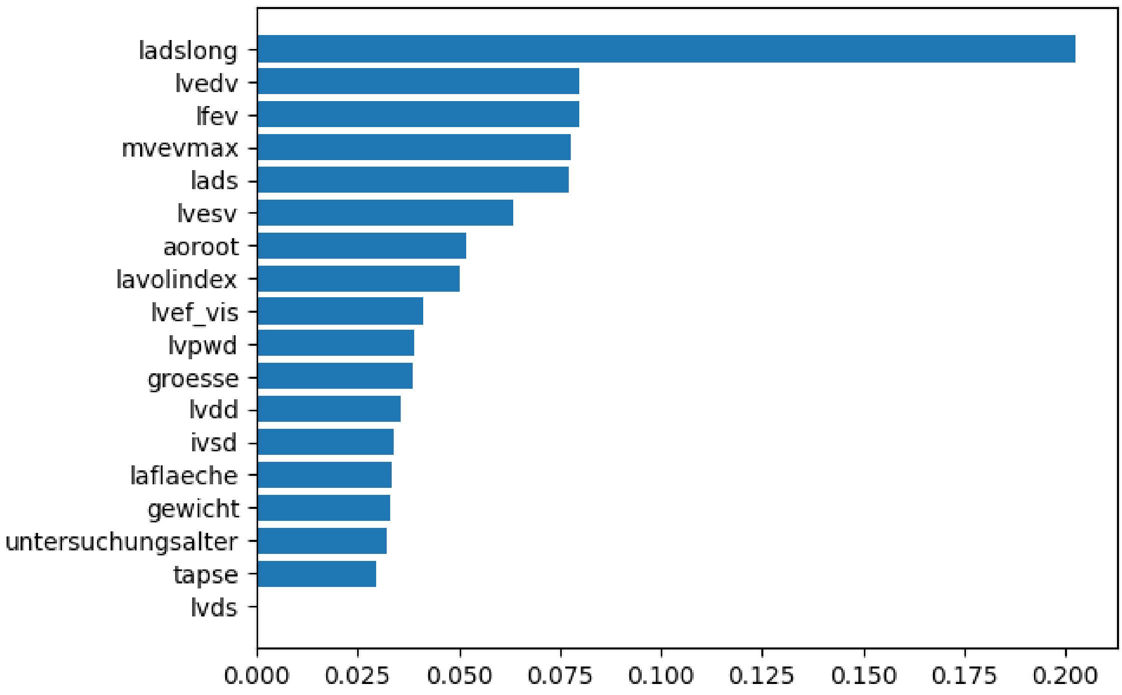

Figure 16. XGB Relevance of Features of the echocardiographic dataset

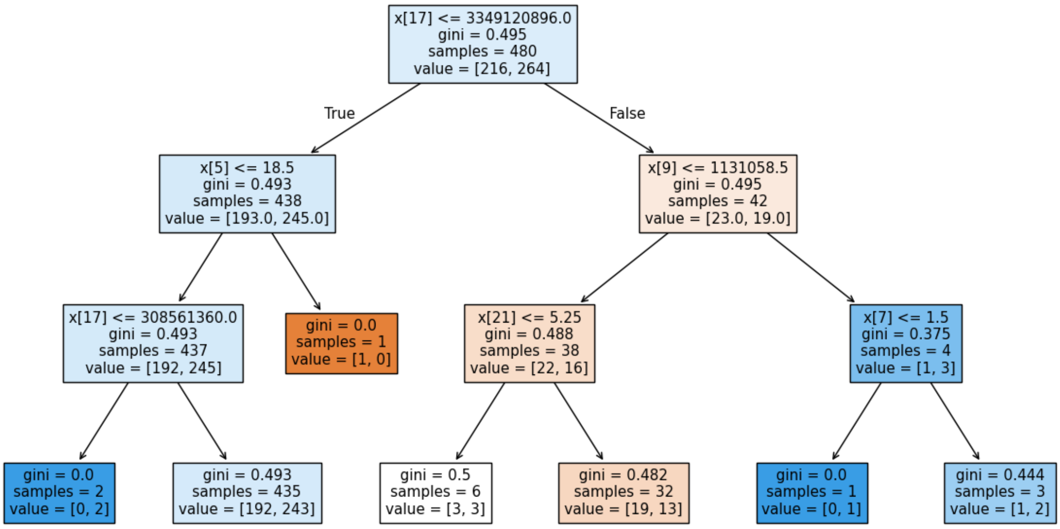

Figure 17. Results of the DT of the medication dataset

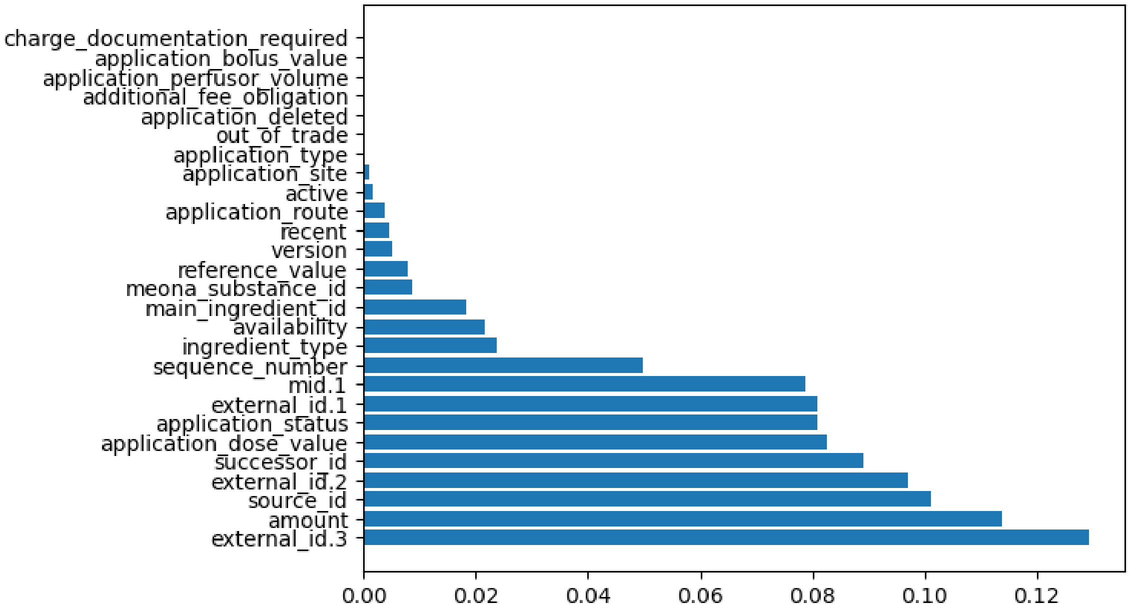

Figure 18. Random Forest Relevance of Features of the medication dataset

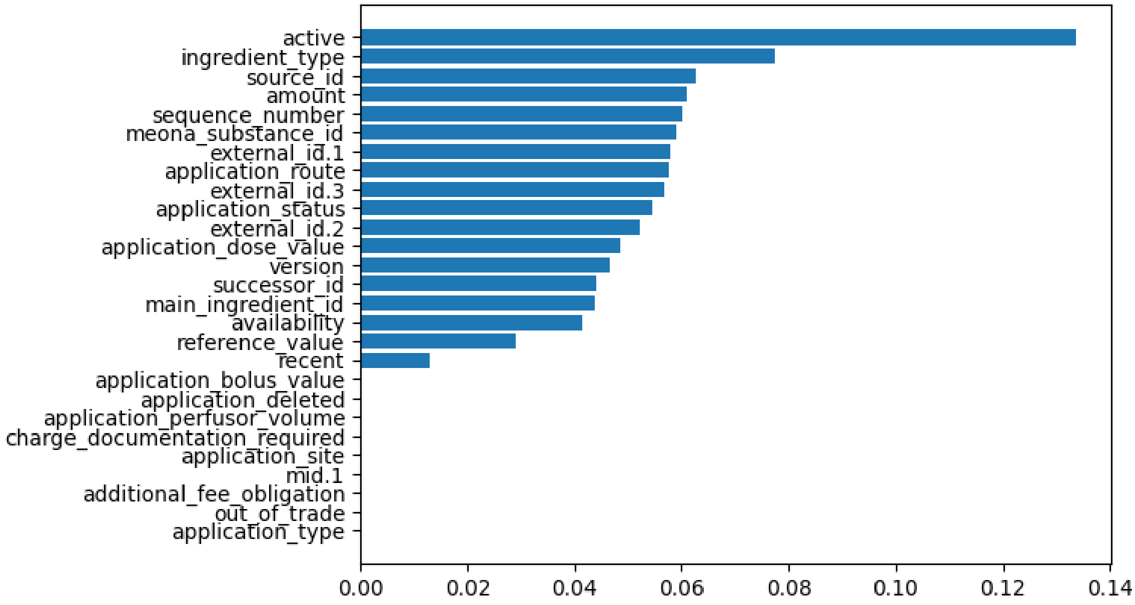

Figure 19. XGB Relevance of Features of the medication dataset
