# Supplementary figures and images for: Proposal for Using AI to Assess Clinical Data Integrity and Generate Metadata: Algorithm Development and Validation
Source: JMIR Med Inform. 2025 Jun 30;13:e60204. doi: 10.2196/60204 (PMC12234397; doi:10.2196/60204)

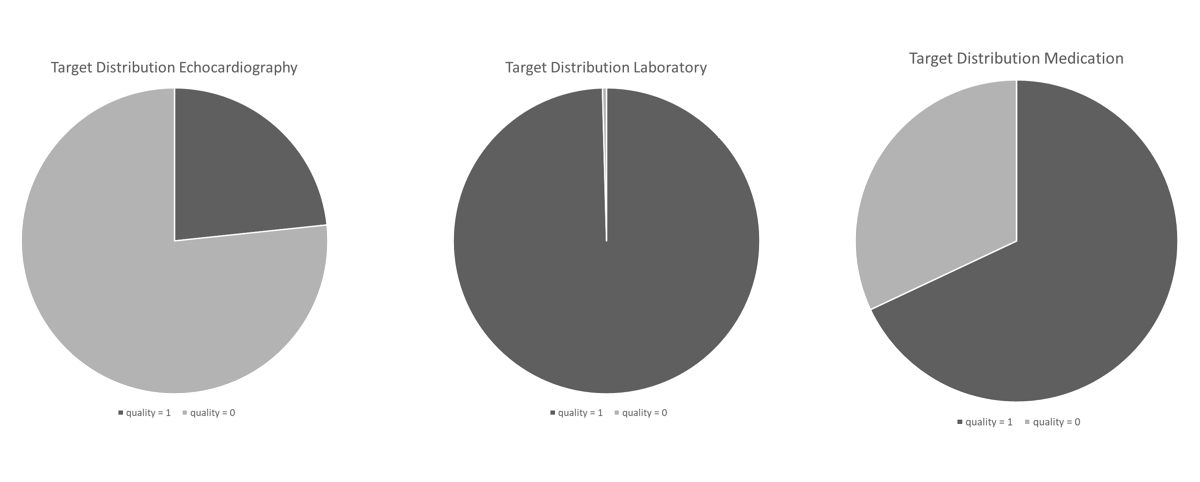

Supplement: Multimedia Appendix 2 [file medinform-v13-e60204-s002.docx]
